# The Gαh/phospholipase C-δ1 interaction promotes autophagosome degradation by activating the Akt/mTORC1 pathway in metastatic triple-negative breast cancer

**DOI:** 10.18632/aging.103390

**Published:** 2020-07-01

**Authors:** Hui-Yu Lin, Chia-Hao Kuei, Hsun-Hua Lee, Che-Hsuan Lin, Jing-Quan Zheng, Hui-Wen Chiu, Chi-Long Chen, Yuan-Feng Lin

**Affiliations:** 1 Graduate Institute of Clinical Medicine, College of Medicine, Taipei Medical University, Taipei, Taiwan; 2 Department of Breast Surgery and General Surgery, Division of Surgery, Cardinal Tien Hospital, Xindian District, New Taipei, Taiwan; 3 Department of Urology, Division of Surgery, Cardinal Tien Hospital, Xindian District, New Taipei, Taiwan; 4 Department of Neurology, Shuang Ho Hospital, Taipei Medical University, New Taipei, Taiwan; 5 Department of Neurology, School of Medicine, College of Medicine, Taipei Medical University, Taipei, Taiwan; 6 Department of Neurology, Vertigo and Balance Impairment Center, Shuang Ho Hospital, Taipei Medical University, New Taipei, Taiwan; 7 Department of Otolaryngology, School of Medicine, College of Medicine, Taipei Medical University, Taipei, Taiwan; 8 Department of Otolaryngology, Taipei Medical University Hospital, Taipei Medical University, Taipei, Taiwan; 9 Department of Critical Care Medicine, Shuang Ho Hospital, Taipei Medical University, New Taipei, Taiwan; 10 Division of Nephrology, Department of Internal Medicine, Shuang Ho Hospital, Taipei Medical University, New Taipei, Taiwan; 11 Department of Pathology, College of Medicine, Taipei Medical University, Taipei, Taiwan; 12 Department of Pathology, Taipei Medical University Hospital, Taipei Medical University, Taipei, Taiwan; 13 Cell Physiology and Molecular Image Research Center, Wan Fang Hospital, Taipei Medical University, Taipei, Taiwan

**Keywords:** gαh, autophagy, Akt/mTORC1, metastasis, triple-negative breast cancer

## Abstract

Lung metastasis (LM) is commonly found in triple-negative breast cancer (TNBC); however, the molecular mechanism underlying TNBC metastasis to lungs remains largely unknown. We thus aimed to uncover a possible mechanism for the LM of TNBC. Here we show that the phosphorylation of Akt and mTORC1 was positively but the autophagy activity was negatively correlated with endogenous Gαh levels and cell invasion ability in TNBC cell lines. Whereas the knockdown of Gαh, as well as blocking its binding with PLC-δ1 by a synthetic peptide inhibitor, in the highly invasive MDA-MB231 cells dramatically suppressed Akt/mTORC1 phosphorylation and blocked autophagosome degradation, the overexpression of Gαh in the poorly invasive HCC1806 cells enhanced Akt/mTORC1 phosphorylation but promoted autophagosome degradation. The pharmaceutical inhibition of autophagy initiation by 3-methyladenine was found to rescue the cell invasion ability and LM potential of Gαh-silenced MDA-MB231 cells. In contrast, the inhibition of mTORC1 activity by rapamycin suppressed autophagosome degradation but mitigated the cell invasion ability and LM potential of Gαh-overexpressing HCC1806 cells. These findings demonstrate that the induction of autophagy activity or the inhibition of Akt-mTORC1 axis provides a useful strategy to combat the Gαh/PLC-δ1-driven LM of TNBC.

## INTRODUCTION

Triple negative breast cancer (TNBC) is defined by a lack of estrogen receptor (ER), progesterone receptor (PR) and human epidermal growth factor 2 receptor (HER2) and remains the most challenging breast cancer to treat. Recently, in accordance with *ESR1, PGR*, and *ERBB2* expression and distinct patterns of molecular alterations, TNBC has been further subcategorized into 7 different subtypes: basal-like 1 (BL1), basal-like 2 (BL2), mesenchymal (M), immunomodulatory (IM), luminal androgenic receptor (LAR), mesenchymal stem-like (MSL) [1]. This seven-subtype classification has been shown to independently predict a pathologic complete response (pCR) but not distant metastasis-free or overall survival in a retrospective analysis of TNBC patients treated with neoadjuvant chemotherapy [2]. Clinically, the life-threatening metastatic spread of TNBC preferentially to the lungs and brain usually occurs within 3 years after surgery and leads to a worse disease-specific outcome than other breast cancer subtypes [3]. In the past decade, major efforts have been made to classify TNBC into distinct clinical and molecular subtypes to effectively guide treatment decisions, prevent the development of metastatic disease and ultimately improve survival in this patient population [4]. However, the molecular mechanism underlying TNBC metastasis remains largely unknown.

Gαh is also known as tissue transglutaminase (tTG) or transglutaminase 2 (TG2) because of its transamidation activity when the ratio of the intracellular Ca^2+^ concentration to the GTP concentration is increased [5]. An increased level of Gαh has been detected in various types of cancer cells and is associated with cancer progression, e.g., therapeutic resistance and metastasis, and poor prognosis [6–11]. Intriguingly, recent reports demonstrated that GTP-binding activity of Gαh, but not transamidation, is required for the metastatic progression of breast cancer [12, 13], although Gαh expression levels are causally correlated with the metastatic potential of other cancers [14, 15]. Our recent report also showed that the coupling of Gαh with phospholipase C-δ1 (PLC-δ1)-related signaling pathway enhances the lung metastasis of TNBC cells [16]. On the other hand, the association between Gαh activity/expression and Akt/mTOR pathway, as well as autophagosome degradation, has been demonstrated in several types of cancer cells [17–22]. Nevertheless, the involvement of the Akt/mTOR pathway and autophagy activity in Gαh/PLC-δ1-driven TNBC metastasis remains unclear.

To this end, in this study, we performed an *in silico* experiment using gene set enrichment analysis (GSEA) of the transcriptional coexpression status of Gαh in primary tumors derived from ER-negative breast cancer patients defined as having low-level Gαh expression without lung metastasis or high-level Gαh expression with lung metastasis. The GSEA results revealed that the mTORC1-related pathway might be activated in the Gαh-associated lung metastasis of ER-negative breast cancer. We also found that the interruption of the Gαh and PLC-δ1 interaction suppresses the activation of Akt/mTORC1 but promotes the initiation of autophagy, which ultimately inhibits the metastatic progression of TNBC cells *in vitro* and *in vivo*. In addition to describing the PPI inhibitor of the Gαh/PLC-δ1 complex, this study suggests another strategy for using a mTORC1 inhibitor, e.g., rapamycin, to combat metastatic TNBC with upregulated Gαh.

## RESULTS

### The upregulation of Gαh accompanied by mTORC1 activation correlates with an increased risk for lung metastasis in ER(-) breast cancer patients

**Figure 1:**
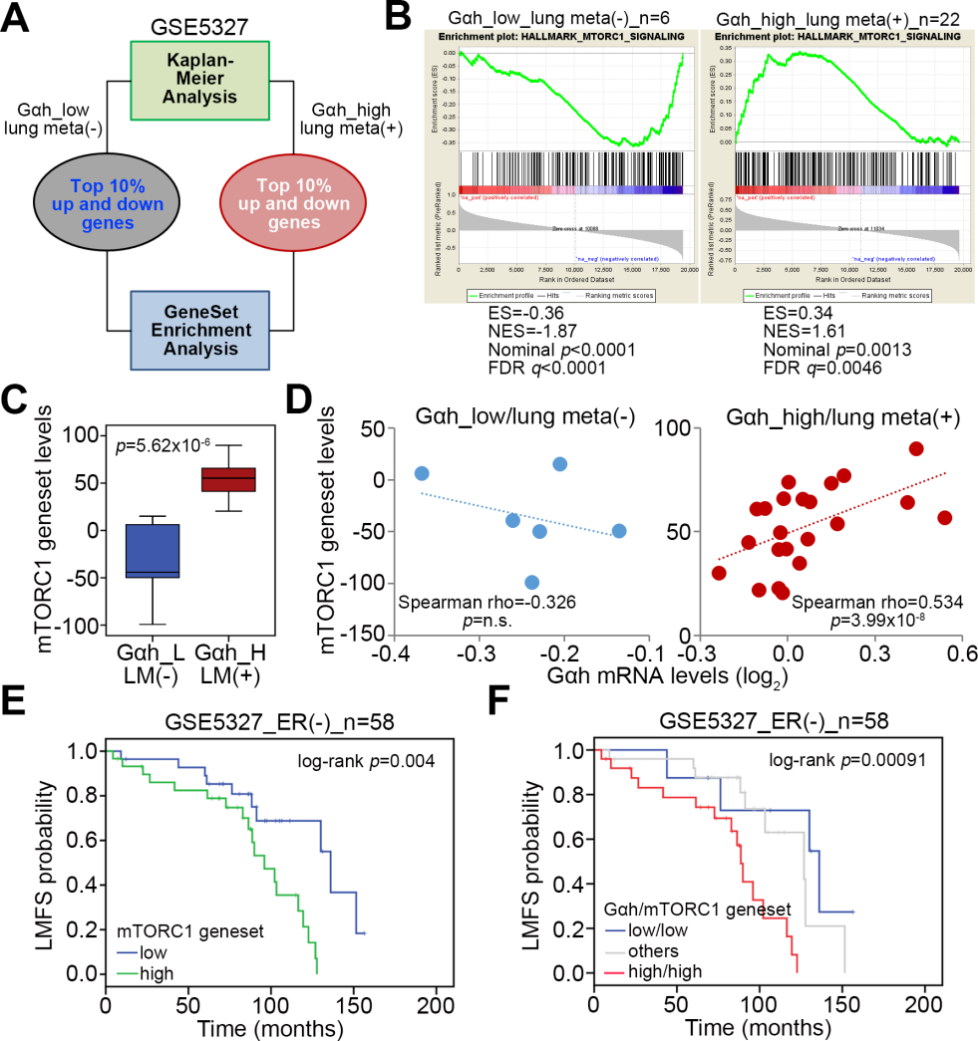
**The mTORC1-related pathway is putatively activated in ER(-) breast cancer with high-level Gαh expression and lung metastasis.** (**A**) Flowchart of the gene enrichment analysis (GSEA) using the transcription profiling of the top 10% upregulated and downregulated genes in ER(-) breast cancer tissues that were defined with low-level Gαh expression without lung metastasis or high-level Gαh expression with lung metastasis in a Kaplan-Meier analysis based on the GSE5327 data set. (**B**) The enrichment score (ES) derived from the correlation between the MTORC1 gene set and the queried gene signatures was plotted as the green curve. The parameters including the normalized enrichment score (NES), nominal p values and false discovery rates (FDRs) are shown as inserts. (**C**) Transcriptional profiling of the MTORC1 gene set in the groups is shown in A. The statistical significance was analyzed by Student’s t-test. (**D**) Correlation of the expression of Gαh mRNA levels and MTORC1 gene in the groups is shown in **A**. (**E** and **F**) Results from the Kaplan-Meier analyses of the transcriptional levels of the mTORC1 gene set alone (**E**) or combined with the mRNA levels of Gαh (**F**) against ER(-) breast cancer patients from the GSE5327 data set.

We selected the top 10% of the upregulated and downregulated genes derived from the non-lung metastatic and lung metastatic ER(-) breast cancer tissues with low- and high-levels of Gαh as previously defined with a Kaplan-Meier analysis [16] to perform an *in silico* gene set enrichment analysis (GSEA) (Figure 1A). GSEA results demonstrated that the MTORC1 signaling pathway is significantly predicted to be inhibited in non-lung metastatic ER(-) breast cancer tissues with low levels of Gαh expression (p<0.01) but activated in lung metastatic ER(-) breast cancer tissues with high levels of Gαh expression (Figure 1B). Accordingly, the number of transcript for the mTORC1 gene set of lung metastatic ER(-) breast cancer tissues with high Gαh levels was prominently higher than the number of the mTORC1 gene sets for non-lung metastatic ER(-) breast cancer tissues with low Gαh levels (Figure 1C). Whereas the mRNA levels of the mTORC1 gene set and Gαh appeared to be negatively correlated in the non-lung metastatic ER(-) breast cancer tissues, their expression levels were significantly and positively correlated in the lung metastatic ER(-) breast cancer tissues with high Gαh levels (p<0.0001) (Figure 1D). The results from the Kaplan-Meier analysis revealed that higher mRNA levels of the mTORC1 gene set correlated with a poor lung metastasis-free survival probability in ER(-) breast cancer patients of the GSE5327 data set (Figure 1E). Moreover, the signature of the combined high-level mTORC1 gene set and Gαh significantly predicted a shortened period for lung metastasis in ER(-) breast cancer patients of the GSE5327 dataset (p=0.00091) (Figure 1F).

### The activity of the Akt/mTOR axis is highly correlated with Gαh levels and cell invasion ability and is most likely regulated by the Gαh-PLC-δ1 interaction in TNBC cells

**Figure 2:**
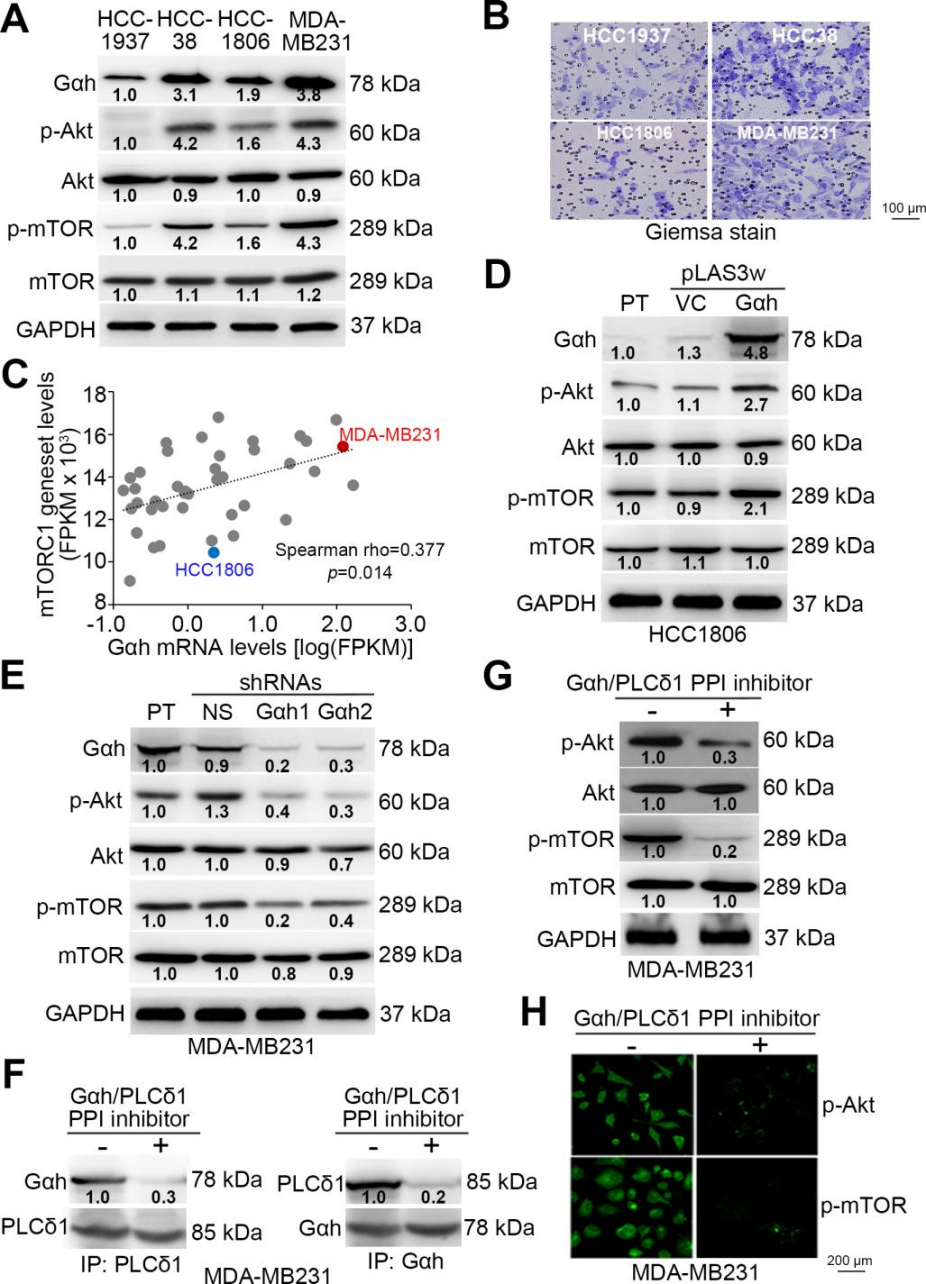
**The phosphorylation of Akt and mTORC1 positively correlates with cell invasion ability and is regulated by the Gαh-PLC-δ1 pathway in TNBC cells.** (**A**) Results from the western blot analysis for the Gαh, phosphorylated Akt (p-Akt), Akt, p-mTOR, mTOR and GAPDH proteins derived from the indicated TNBC cell lines. (**B**) Giemsa staining of the invaded cells of the tested TNBC cell lines after a 16-hour invasion assay. (**C**) Correlation of mRNA expression levels between Gαh and the mTORC1 gene set in a panel of breast cancer cell lines derived from the Cancer Cell Line Encyclopedia (CCLE) database. Spearman’s correlation test was used to estimate the statistical significance. (**D**–**E**) Results from the western blot analysis for the Gαh, p-Akt, Akt, p-mTOR, mTOR and GAPDH proteins derived from the parental (PT) HCC1806 cells without (vector control, VC) or with Gαh overexpression (**D**) and the parental MDA-MD231 cells without (nonsilenced, NS) or with Gαh knocked down using two independent shRNA clones (**E**). (**F**–**H**) MDA-MD231 cells treated without or with 10 μM Gαh/PLC-δ1 protein-protein interaction (PPI) inhibitor for 2 hours were subjected to a reciprocal immunoprecipitation for detecting the PPI of Gαh/PLC-δ1 (**F**), Western blot analysis for measuring the protein levels of p-Akt, Akt, p-mTOR, mTOR and GAPDH (**G**), and immunofluorescent staining for visualizing the intracellular protein levels of p-Akt and p-mTOR (**H**). In **A**, **D**, **E**, **G**, GAPDH was used as an internal control of protein loading. The protein intensities of representative blots from three independent experiments were normalized by GAPDH levels and presented as a ratio to the control group.

Since mTORC1 activation is tightly regulated by the Akt protein kinase through the modulation of protein phosphorylation, we examined the phosphorylated protein levels of Akt and mTORC1 in a panel of TNBC cell lines (Figure 2A). The data showed that the levels of the phosphorylated Akt and mTORC1 proteins are prominently higher in MDA-MB231 cells, which exhibit a strong invasive ability (Figure 2A and 2B). In contrast, the levels of phosphorylated Akt and mTORC1 appeared to be relatively low in the poorly invasive HCC1806 cells (Figure 2A and 2B). By using RNA sequencing results for a panel of TNBC cell lines deposited in TCGA database, we found that the association between the mRNA levels of the mTORC1 gene set and Gαh was significantly positive (p=0.014) (Figure 2C). These data validated our finding that the levels of Gαh and activated Akt/mTORC1 are causally associated with the invasive abilities of MDA-MB231 and HCC1806 cells (Figure 2A–2C). To confirm that Gαh expression modulates the activity of the Akt/mTORC1 signaling axis, we performed experiments with Gαh overexpression and knockdown. Whereas the overexpression of the exogenous Gαh gene dramatically elevated the levels of phosphorylated Akt and mTORC1 (Figure 2D) and eventually enhanced the invasion ability of HCC1806 cells [16], artificially silencing the expression of endogenous Gαh prominently suppressed the levels of phosphorylated Akt and mTORC1 (Figure 2E) and ultimately mitigated the invasion ability of MDA-MB231 cells [16]. Since the coupling of Gαh with phospholipase C-δ1 (PLC-δ1) has been previously shown to promote metastatic progression in TNBC [16], we determined whether the activity of the Akt/mTORC1 signaling axis is regulated by the interaction between Gαh and PLC-δ1. Treatment with the protein-protein interaction (PPI) inhibitor of the Gαh/PLC-δ1 complex predominantly interrupted the PPI of Gαh/PLC-δ1 complex (Figure 2F) and robustly abolished the levels of constitutively phosphorylated Akt and mTORC1 (Figure 2G–2H) and consequently diminished the invasion ability of the highly invasive MDA-MB231 cells [16].

### The coupling of Gαh/PLC-δ1 with Akt/mTORC1 promotes autophagosome degradation to promote the metastatic potential of TNBC cells

**Figure 3:**
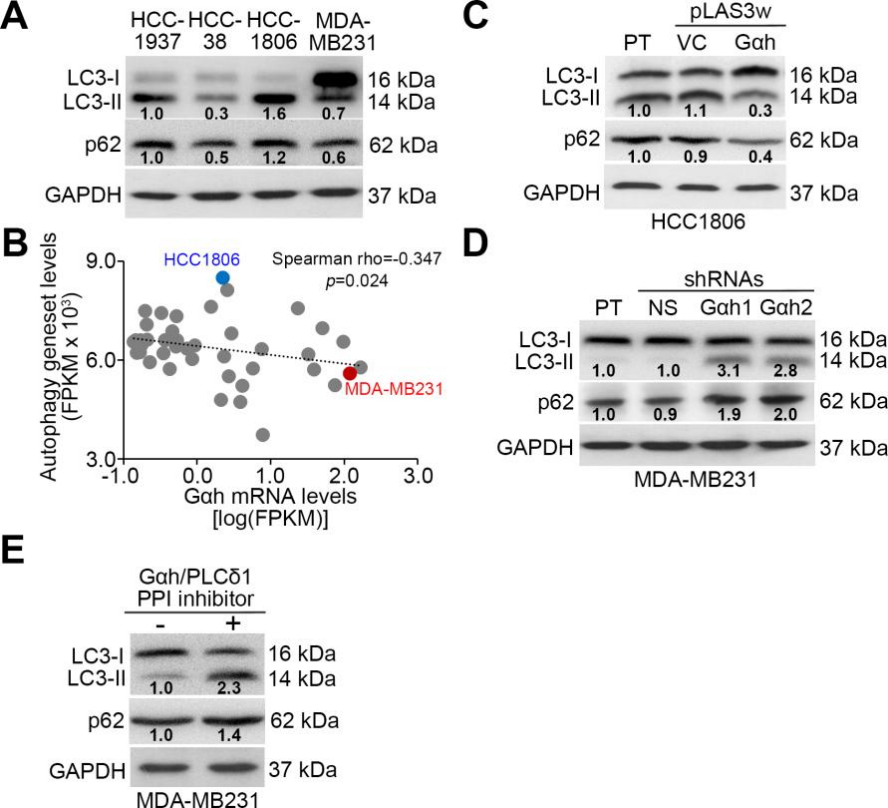
**The Gαh-PLC-δ1 axis promotes autophagosome degradation in TNBC cells.** (**A**) Results from the Western blot analysis for LC3-I/II, p62 and GAPDH proteins derived from the indicated TNBC cell lines. (**B**) Correlation of mRNA expression levels of Gαh and the autophagy-related gene set in a panel of breast cancer cell lines derived from the Cancer Cell Line Encyclopedia (CCLE) database. Spearman’s correlation test was used to estimate the statistical significance. (**C**–**E**) Results from the Western blot analysis of the LC3-I/II, p62 and GAPDH proteins derived from the parental (PT) HCC1806 cells without (vector control, VC) or with Gαh overexpression (**C**) and the parental MDA-MD231 cells without (nonsilenced, NS) or with Gαh knocked down using two independent shRNA clones (**D**), and MDA-MD231 cells treated without or with 10 μM Gαh/PLC-δ1 protein-protein interaction (PPI) inhibitor for 2 hours (**E**). In **A**, **C**, **D**, **E**, GAPDH was used as an internal control of protein loading. The protein intensities of representative blots from three independent experiments were normalized by GAPDH levels and presented as a ratio to the control group.

Since the Akt/mTORC1 signaling axis has been shown to promote autophagosome degradation [20], we examined whether autophagosome assembly is involved in Gαh/PLC-δ1-modulated metastatic progression in TNBC. Results from the Western blot analysis revealed that the endogenous levels of LC3-II, which is a phosphatidylethanolamine-conjugated LC3-I and thought to be involved in autophagosome membrane expansion and fusion events, in the poorly invasive HCC1806 cells were higher than they were in the highly invasive MDA-MB231 cells (Figure 3A). Similar view was also found in the endogenous levels of p62, one of autophagy-specific substrate (Figure 3A). Moreover, the mRNA levels between the autophagy-related gene set that was generated to estimate the autophagy activity and Gαh were negatively correlated in a panel of TNBC cell lines (Figure 3B). Whereas the highly invasive MDA-MB231 cells had a signature of a high level of Gαh expression and a low level of autophagy-related gene set expression, the poorly invasive HCC1806 cells had a signature of low-level Gαh expression but a high level of autophagy-related gene set expression (Figure 3B). The overexpression of the exogenous Gαh gene in the poorly invasive HCC1806 cells reduced the intracellular LC3-II and p62 protein levels (Figure 3C). In contrast, knocking down Gαh increased the intracellular LC3-II and p62 protein levels (Figure 3D). The addition of a PPI inhibitor against the Gαh/PLC-δ1 complex promoted the formation of LC3-II and increased the p62 protein levels in the highly invasive MDA-MB231 cells (Figure 3E).

### Autophagosome degradation positively regulates the Gαh-enhanced metastatic potential in TNBC cells

**Figure 4:**
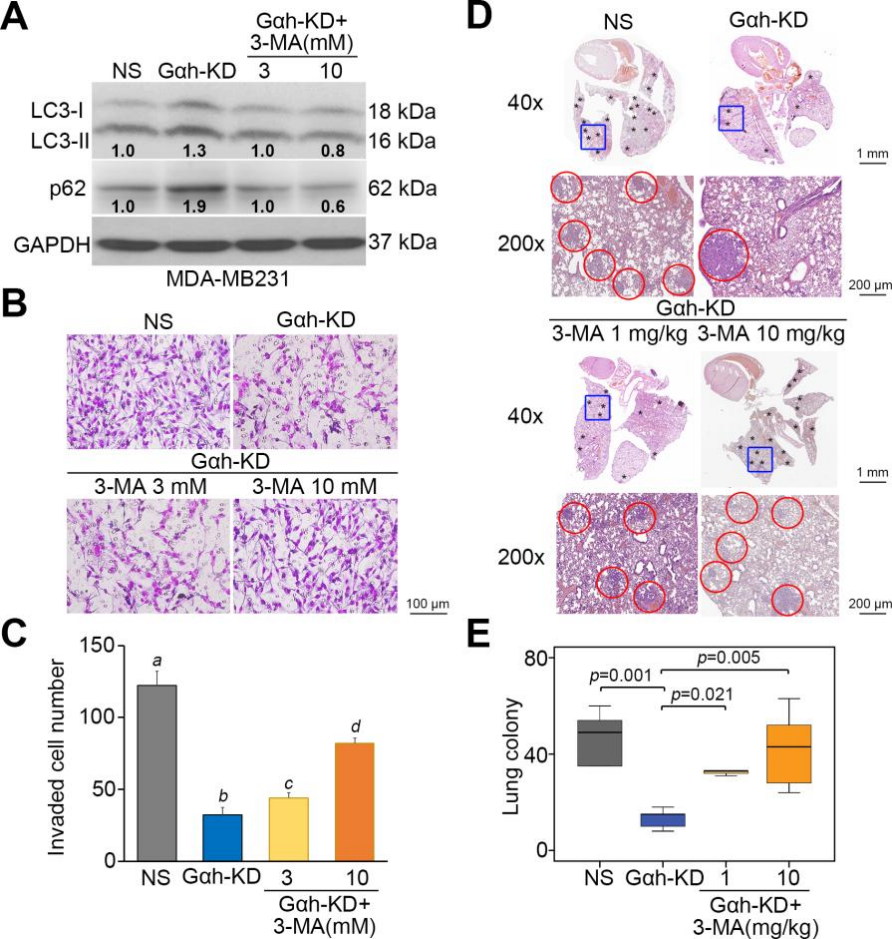
**The inhibition of autophagy initiation by 3-MA rescues the metastatic potential of the Gαh-silenced MDA-MB231 cells *in vitro* and *in vivo*.** (**A**) The results from the Western blot analysis for the LC3-I/II, p62 and GAPDH proteins derived from MDA-MD231 cells without (NS) or with Gαh knocked down (KD) in the absence or presence of the autophagy inhibitor 3-MA (3 or 10 mM). GAPDH was used as an internal control of protein loading. The protein intensities of representative blots from three independent experiments were normalized by GAPDH levels and presented as a ratio to the control group. (**B**–**C**) Giemsa staining (**B**) and cell number (**C**) of the invaded MDA-MD231 cell variants shown in A. Data obtained from three independent experiments are presented as the mean ± SEM. Letters indicate the significant differences at *p*<0.01 analyzed by nonparametric Friedman test. (**D** and **E**) H&E stained lung tissues (**D**) and the number of lung tumor colonies tumors (**E**) derived from the mice (n=5) transplanted with MDA-MD231 cell variants, shown in A, through tail vein injection for 4 weeks. Tumors are shown in red circles. Statistical significance was determined by nonparametric Mann-Whitney U test.

**Figure 5:**
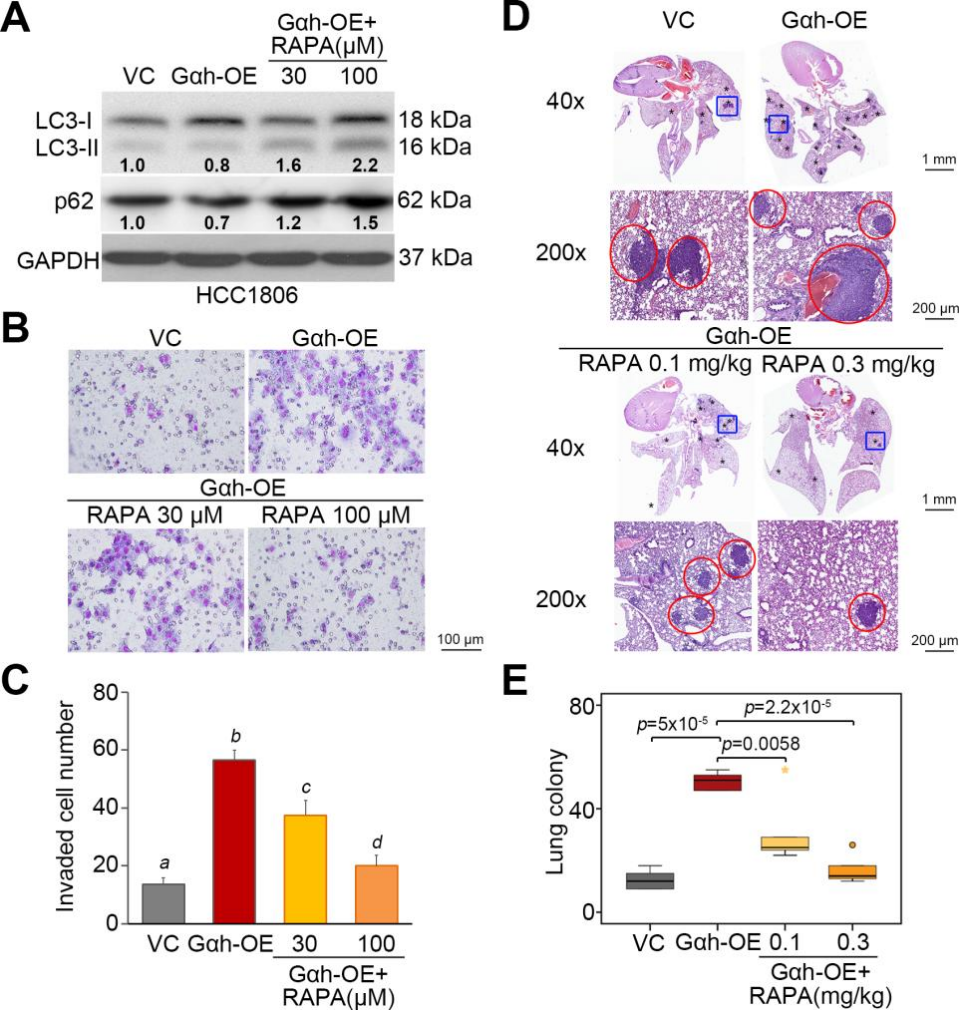
**The inhibition of mTORC1 activity by rapamycin restores autophagy function but compromises the cellular invasion and lung metastatic abilities of Gαh-overexpressing HCC1806 cells.** (**A**) Results from the Western blot analysis of the LC3-I/II, p62 and GAPDH proteins derived from HCC1806 cells without (VC) or overexpression Gαh (OE) in the absence or presence of the mTOR inhibitor rapamycin (RAPA) (30 or 100 μM). GAPDH was used as an internal control of protein loading. The protein intensities of representative blots from three independent experiments were normalized by GAPDH levels and presented as a ratio to the control group. (**B**–**C**) Giemsa staining (**B**) and cell number (**C**) for the invaded HCC1806 cell variants shown in A. Data obtained from three independent experiments are presented as the mean ± SEM. Letters indicate the significant differences at *p*<0.01 analyzed by nonparametric Friedman test. (**D** and **E**) H&E staining of lung tissues (**D**) and the number of lung tumor colonies (**E**) derived from mice (n=5) transplanted with the HCC1806 cell variants, shown in A, through tail vein injection for 4 weeks. Tumors are shown in red circles. Statistical significance was analyzed by nonparametric Mann-Whitney U test.

To realize the critical role of autophagosome degradation in Gαh-promoted metastatic progression in TNBC cells, the autophagy inhibitor 3-methyladenine (3-MA) and the mTORC1 inhibitor rapamycin (RAPA) were used to suppress autophagy initiation in Gαh-silenced MDA-MB231 cells and Gαh-overexpressing HCC1806 cells. Treatment with 3-MA suppressed the LC3-II and p62 protein levels enhanced by Gαh knockdown (Figure 4A) and ultimately rescued the invasive ability (Figure 4B and 4C) of the MDA-MB231 cells. Accordingly, the administration of 3-MA into tumor-bearing mice restored the lung colony forming ability in a dose-dependent manner that had been prominently suppressed after Gαh was knocked down in MDA-MB231 cells (Figure 4D and 4E). Conversely, the introduction of RAPA forced LC3-II and p62 protein levels (Figure 5A) and eventually diminished the cellular invasive ability (Figure 5B and 5C) of Gαh-overexpressing HCC1806 cells. Similarly, treatment with RAPA significantly (p<0.01) suppressed the colony formation of Gαh-overexpressing HCC1806 cells in the lungs of tumor-bearing mice (Figure 5D and 5E).

### The signature of the combination of high-level Gαh and low-level autophagy-related gene set expression predicts a higher risk for lung metastasis in ER(-) breast cancer patients

**Figure 6:**
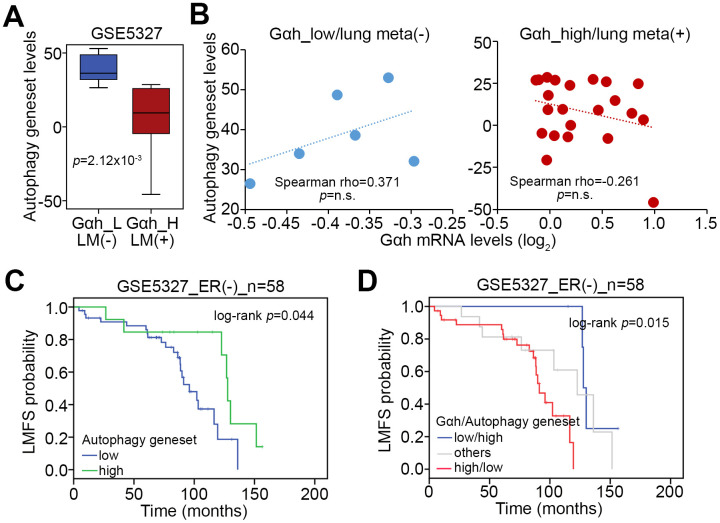
**The signature of the combined Gαh upregulation and low levels of autophagy activity increases the likelihood of lung metastasis in ER(-) breast cancer patients.** (**A**) Transcriptional profiling of the autophagy-related gene set in the groups shown in Figure1A. The statistical significance was analyzed by Student’s t-test. (**B**) Correlation of the expression levels of Gαh mRNA and the autophagy-related gene set in the stratified groups. (**C** and **D**) Results from the Kaplan-Meier analyses for the transcriptional level of the autophagy-related gene set alone (G) or combined with the mRNA level of Gαh (**D**) against ER(-) breast cancer patients from the GSE5327 data set.

We next analyzed the transcriptional profiling of the autophagy gene set in the GSE5327 data set. The data showed that the expression levels of the autophagy-related gene set in the primary tumors derived from ER(-) breast cancer patients who are positive for lung metastasis and in the group with high levels of Gαh on the basis of the Kaplan-Meier analysis [16] were much lower than those of ER(-) breast cancer patients without lung metastasis and in the group with low levels of Gαh (Figure 6A). Whereas the mRNA levels of Gαh and the autophagy-related gene set were positively correlated in the primary tumors derived from the cohort defined as having low levels of Gαh and being negative for lung metastasis, the levels of mRNA expression of Gαh and autophagy-related genes were found to be negatively correlated in primary tumors derived from the cohort defined as having high levels of Gαh and being positive for lung metastasis (Figure 6B). The results from the Kaplan-Meier analyses demonstrated that the low expression levels of the autophagy-related gene set were related to poor prognosis for lung metastasis-free survival of ER(-) breast cancer patients (Figure 6C). Moreover, the signature of the combination of a high level of Gαh expression and a low level of autophagy-related gene set expression was significantly correlated to an unfavorable risk for lung metastasis in ER(-) breast cancer patients (p=0.015) (Figure 6D).

## DISCUSSION

Recent reports have demonstrated that the activation of the Akt/mTOR pathway induced by the long noncoding RNAs OECC [23] and MetaLnc9 [24] and the transmembrane 7 superfamily member 4 [25] promotes metastatic progression; conversely, the suppression of the Akt/mTOR pathway in the presence of the ferulic acid derivative FXS-3 [26], cardamonin [27] and microRNA-520a-3p [28] inhibits the metastatic potential of lung cancer cells. Accordingly, the association of the Akt/mTOR pathway with metastatic progression has been reported in other cancer types, including colorectal cancer [29–31], hepatocellular carcinoma [32–34], endometrial cancer [35, 36], ovarian cancer [37], gastric cancer [38–40], melanoma [41], glioma [42, 43], pancreatic ductal adenocarcinoma [44], nasopharyngeal carcinoma [45, 46], osteosarcoma [47–50], renal cell carcinoma [51–53] and prostate cancer [54–56]. In breast cancer, synaptopodin-2 [57] and caveolin-1 [58] have been shown to modulate Akt/mTOR-regulated metastatic progression. Particularly in TNBC, the inclusion of the Rhizoma Amorphophalli appeared to inhibit cell migration, invasion and metastasis by suppressing the Akt/mTOR pathway [59]. Here, we also found that the Akt/mTOR pathway mediates Gαh-promoted TNBC metastatic progression. However, further experiments are still needed to explore a comprehensive scenario in which the Gαh-PLC-δ1 interaction directly activates the Akt/mTOR pathway in TNBC cells.

The role of autophagy during cancer metastasis is still controversial. A recent review article indicated that autophagy is upregulated during cancer metastasis [60]. In contrast, several lines of evidence have illustrated that autophagy is suppressed during the metastatic progression of some cancer types [61–64]. In TNBC cells, e.g., MDA-MB-231 cells, the induction of autophagy by the selenopurine molecule SLLN-15 has been shown to suppress the metastatic potential *in vitro* and *in vivo* by inhibiting Akt-mTORC1 signaling [65]. Accordingly, treatment with parthenolide, a sesquiterpene lactone found in *Tanacetum parthenium*, appeared to generate autophagy and ultimately suppress the lung metastasis of MDA-MB231 cells in an orthotopic mouse model of breast cancer [66]. Conversely, in an orthotopic mouse model of breast cancer, the suppression of autophagy via hypoxia-induced expression of the kinase-dead unc-51-like autophagy-activating kinase (ULK1) mutant K46N was found to increase the lung metastasis capacity of MDA-MB-231 cells [67]. Here, we also found that the induction of autophagy by rapamycin treatment suppresses the metastatic potential of Gαh-overexpressing HCC1806 cells, whereas the inhibition of autophagy by 3-MA treatment restores the metastatic capacity of Gαh-silenced MDA-MB-231 cells. These findings may elucidate a negative role of autophagy in regulating TNBC metastasis.

Because the Akt/mTOR pathway is one of the important pathways involved in TNBC progression, several Akt/mTOR inhibitors used as monotherapy or in combination therapy for TNBC patients are currently in phase I/II clinical trials [68]. Moreover, the therapeutic targeting of autophagy activity has also been thought to be another promising anticancer strategy. Therefore, our results provide a new strategy to combat the metastatic progression of TNBC due to Gαh upregulation via inhibiting Akt/mTOR activity or preventing autophagosome degradation.

## MATERIALS AND METHODS

### Cell lines and cell culture conditions

MDA-MB231 cells were cultured in Leibovitz’s (L-15) medium (Invitrogen) supplemented with 10% fetal bovine serum (FBS, Invitrogen). HCC1937, HCC1806, and HCC38 cells were cultured in RPMI 1640 medium (Invitrogen) with 10% FBS. 293T cells were cultured in DMEM medium with 10% FBS. All cell lines were obtained from American Type Culture Collection (ATCC). All cells were incubated at 37°C with 5% CO_2_ and routinely authenticated on the basis of short tandem repeat (STR) analysis, morphologic and growth characteristics of the cells and mycoplasma detection.

### Microarray data processing

Microarray data and related clinical data from the Gene Expression Omnibus (GEO) GSE5327 data set were downloaded from the NCBI website. Affymetrix DAT files were processed using the Affymetrix Gene Chip Operating System (GCOS) to generate .CEL files. Raw intensities in the .CEL files were normalized by robust multichip analysis (RMA), and fold-change analysis was performed using GeneSpring GX11 (Agilent Technologies). Relative mRNA expression levels were normalized by their median and presented as log_2_ values. The gene set of autophagy was obtained from Molecular Signatures Database (https://www.gsea-msigdb.org/gsea/msigdb). The sum derived from the expression levels of the gene set was used to represent the autophagy activity.

### Plasmid construction, preparation and infection of lentiviral particles

The gene that encodes Gαh was amplified from human cDNA (Invitrogen), using the standard polymerase chain reaction (PCR) procedure with paired primers, and subcloned into pLAS3w/Ppuro according to the procedure described in our previous report [16]. All lentiviral vectors, including pLAS3w/Ppuro and the derivatives of a shRNA vector, were obtained from the National RNAi Core Facility Platform in Taiwan. All vectors were cotransfected with the pMD. G and pCMVΔR8.91 plasmids using a calcium phosphate transfection kit (Invitrogen) into 293T cells. After 48 hours of incubation, the viral supernatants were collected and transferred to the target cells, and then, the infected cells were cultured in the presence of puromycin (Calbiochem) at 5 - 10 μg/ml to select the stably transfected cells.

### Immunoprecipitation and Western blot assay

Whole cell lysates (1 mg) were pre-incubated with non-immunized serum and protein A/G-conjugated agarose (Santa Cruz) for 1 hour at 4°C with a gentle rotation. After the centrifugation, the supernatants were further incubated with Gαh or PLC-δ1 (Gentex) antibodies and protein A/G-conjugated agarose overnight at 4°C with a gentle rotation. After several washes, the immunoprecipitates were resuspended in 20 μl of SDS-PAGE protein loading dye and boiled at 95°C. After the centrifugation, the supernatants were subjected to Western blot analysis.

Total protein (100 μg) from the designed experiments was separated by SDS-PAGE and then transferred to PVDF membranes. The membranes were sequentially incubated with blocking buffer (5% nonfat milk in TBS containing 0.1% Tween-20) for 2 hours at room temperature, primary antibodies against Gαh, PLC-δ1 (Gentex), phosphorylated Akt, Akt, phosphorylated mTOR, mTOR, LC3-I/II and p62 (Cell Signaling) or GAPDH (AbFrontier) overnight at 4 °C, and peroxidase-labeled secondary antibodies for 1 hour at room temperature. At each step, the cells were extensively washed. Finally, immunoreactive bands were visualized by an enhanced chemiluminescence system (Amersham Bioscience).

### Immunofluorescent staining

MDA-MB231 cells (1 x 10^5^/ml) cultivated in the absence or presence of Gαh/PLC-δ1 PPI inhibitor and grown on cover slides (22 mm in diameter and 0.17 mm in thickness) were fixed in 4% formaldehyde for 15 min at RT. After washing cells two times with PBS, the cells were treated with 95% EtOH/5% CH3COOH at-20°C for 15 min. Before blocking with 2% BSA/0.1% Triton X-100 for 2 hours at room temperature (RT), the cells were washed two times with PBS. Subsequently, the cells were incubated with p-Akt or p-mTOR antibody overnight at 4°C. After washing the cells three times with PBS, the cells were incubated with biotin-conjugated secondary antibody (DAKO) for 1 hour at RT. The cells were washed three times with PBS and incubated with fluorescein-conjugated avidin complex (Vector Laboratories) for 30 min at RT. After mounting the cells were analyzed using a FluoView confocal microscope system (Olympus).

### Invasion assay

Cell invasion ability was measured by Boyden Chambers (Neuro Probe) according to the manufacturer’s protocol. Briefly, a polycarbonate membrane (8 μm pore size, 25 × 80 mm, Neuro Probe) was precoated with 10 μg human fibronectin (Sigma) on the lower side and Matrigel (BD Biosciences) on the upper side. The cells (1.5 x 10^4^) obtained from the designed experiments were plated in the top chamber with 50 μl serum-free medium. After 16 hours, stationary cells from the top side of the membrane were removed, whereas the invaded cells in the bottom side of the membrane were fixed in 100% methanol and stained with 10% Giemsa solution (Merck) for 1 hour. The number of invaded cells was counted under a light microscope (400×, ten random fields from each well). All experiments were performed in triplicate.

### Animal experiments

NOD/SCID mice were obtained from the National Laboratory Animal Center in Taiwan and maintained in compliance with institutional policy. All animal procedures were approved by the Institutional Animal Care and Use Committee at Taipei Medical University. For the *in vivo* lung metastatic colonization assay, 1x10^6^ cells in 100 μl PBS were implanted into the mice through tail vein injection. The mice were sacrificed, and the lungs were obtained for histological analysis. Metastatic lung nodules were quantified after staining with H&E using a dissecting microscope.

### Statistical analyses

SPSS 17.0 software (Informer Technologies, Roseau, Dominica) was used to analyze statistical significance. Nonparametric Mann-Whitney U tests were utilized to compare mTORC1 and the autophagy-related gene set expression in breast cancer patients. Spearman’s test was performed to estimate the association among Gαh, mTORC1 and autophagy-related gene set expression levels in breast cancer tissues and in the panel of the TNBC cell lines. Survival probabilities were determined by Kaplan-Meier analysis and log-rank tests. Nonparametric Mann-Whitney U and Friedman tests were used to analyze data from 2 independent samples and 3 or more related samples, respectively. P values <0.05 in all analyses were considered to be statistically significant.
